# Visual Acuity, Retinal Sensitivity, and Macular Thickness Changes in Diabetic Patients without Diabetic Retinopathy after Cataract Surgery

**DOI:** 10.1155/2017/3459156

**Published:** 2017-01-24

**Authors:** Spela Stunf Pukl, Nataša Vidović Valentinčič, Mojca Urbančič, Irena Irman Grčar, Rok Grčar, Vladimir Pfeifer, Mojca Globočnik Petrovič

**Affiliations:** ^1^Eye Hospital, University Medical Center Ljubljana, Ljubljana, Slovenia; ^2^Eye Center Irman, Žalec, Slovenia; ^3^Faculty of Medicine, University of Ljubljana, Ljubljana, Slovenia

## Abstract

*Aim*. Functional and morphological macular study after cataract surgery in a group of diabetics without diabetic retinopathy compared to nondiabetics to evaluate the effect of surgical oxidative stress on diabetic retina.* Methods*. Prospective, comparative study. Preoperative eye exam, best corrected visual acuity (BCVA) measured by ETDRS letters, and optical coherence tomography (OCT) were followed by standard cataract surgery. The follow-up visits at 1, 3, and 6 months postoperatively included BCVA, OCT, and microperimetry, to analyze changes within and between the groups.* Results*. The BCVA improved significantly in diabetics and controls: 64.2 to 81.0 and 61.9 to 82.1 ETDRS at 6 months, respectively. The central macula at OCT significantly thickened in both groups, while the central 5 fields, corresponding to the microperimetry area, subclinically thickened from 284.20 to 291.18 *μ*m at 6 months only in diabetics (*p* = 0.026). A matching slight decrease in the microperimetry sensitivity from 1 to 6 months was found also only in diabetics, with mean average difference −0.75 dB (*p* = 0.04).* Conclusion*. Underlying diabetes does not influence the surgical outcome in diabetics without diabetic retinopathy. However, slight thickening of wider macula and corresponding decrease in retinal sensitivity observed in diabetics 6 months postoperatively might influence visual function on long term.

## 1. Introduction

Hyperglycemia activates several biochemical pathways leading to oxidative stress, the hallmark in the pathogenesis of diabetic retinopathy [1].

The term oxidative stress refers to an imbalance between the antioxidant defense system of the cell and the intracellular amount of harmful reactive oxygen species (ROS). Oxidative stress may result from endogenously produced ROS (caused by hyperglycemia) or by external sources (caused by cataract operation). Cataract surgery influences the intraocular balance in different aspects. It is one of the well-known sources of free radicals [2–6], and it also causes a medium termed lowering of the antioxidative substances in the anterior chamber [7–11].

As a result of free radicals accumulation, the risk of developing or worsening of macular edema following cataract surgery is higher in patients with diabetes than in patients without diabetes and correlates well with the progression of diabetic retinopathy [12–17].

There are some controversies in the results of the studies reporting developed increase in central macular thickness (CMT) or macular edema after cataract surgery in patients with diabetes but no diabetic retinopathy (in diabetic patients without diabetic retinopathy). In a retrospective database study performed on more than 4500 diabetics without preoperative macular edema, the postoperative incidence of macular edema was reported 4%, higher than in the population without diabetes (*p* < 0.001) [14]. The risk for development of macular edema in diabetics without retinopathy (RR 1.80) was reported to be higher than in the population without diabetes (RR 1.17) [14].

On the contrary, a recently published meta-analysis showed no statistically significant increase in CMT values after cataract surgery in diabetic patients without diabetic retinopathy at 1, 3, and 6 months after cataract extraction [12]. On the other hand, Katsimpris et al. found increased macular thickness after uncomplicated cataract surgery in diabetics without retinopathy compared to preoperative values or to a control group of patients at all follow-ups up to twelve months after cataract surgery [18].

This clinical study was designed to compare visual function (visual acuity, retinal sensitivity) and morphologic retinal changes (macular thickness) before and after cataract surgery in diabetic patients without diabetic retinopathy in comparison to nondiabetic patients.

## 2. Methods

### 2.1. Patient Enrolment

This prospective, comparative study was designed to assess the clinical outcome of diabetic patients without diabetic retinopathy undergoing cataract surgery. Participant enrolment and treatment took place at the Eye Hospital, University Medical Center Ljubljana, Slovenia. The study was approved by the Republic of Slovenia's National Medical Ethics Committee and conducted in accordance with the Declaration of Helsinki 1964. Written and fully informed consent was voluntarily provided by all participants prior to enrolment in the clinical study.

Inclusion criteria were clinically significant age related cataract of LOCS III (lens opacities classification system) grade N3, diabetes mellitus type II for the group of diabetic patients, and no diabetes as proved by fasting blood glucose test for the control group.

Exclusion criteria were any other ocular pathology except cataract.

Eighteen eyes of diabetics without diabetic retinopathy and 10 eyes of nondiabetic patients were included in the test and the control group, respectively.

Preoperative visit evaluation was followed by a standard microinvasive cataract surgery. Postoperative follow-up visits were scheduled for day 1, 1 month, 3 months, and 6 months postoperatively.

### 2.2. Cataract Surgery

A standard cataract surgery with phacoemulsification was performed by one of two surgeons (NVV, VP) as follows: preoperatively, topical installation of NSAID (Naclof®, Alcon, Texas) three times in 10-minute intervals, corticosteroid and antibiotic (Maxitrol®, Alcon, Texas) three times in 10-minute intervals, topical mydriatics, a combination of two eye drops, 1% tropicamide (Mydriacyl 1%®, Alcon Pharmaceuticals, Hünenberg, Switzerland) and generic 2,5% phenylephrine, three times in 10-minute intervals, followed by topical anesthetic and iodine application performed in 5-minute intervals. After sterile preparation, a 2,2 mm clear corneal incision, intracameral lidocaine, intracameral hydroxypropyl methylcellulose (Acryvisc®, Zeiss, Oberkochen, Deutschland), and 5–5.5 mm continuous curvilinear capsulorhexis were followed by phacoemulsification performed at the same phacomachine (Millenium®, Bausch & Lomb Storz) in all patients, aspiration irrigation, and hydrophobic IOL implantation. Intracameral antibiotic (1.0 mL generic vancomycin used off-label) was administrated at the end of the surgery. Postoperatively, topical NSAID (Naclof, Alcon, Texas) to prevent cystoid macular edema [19] and corticosteroid and antibiotic (Maxitrol, Alcon, Texas), both three times a day, were prescribed for 3 weeks in both groups.

### 2.3. Preoperative and Postoperative Assessments and Outcome Measures

**Figure 1:**
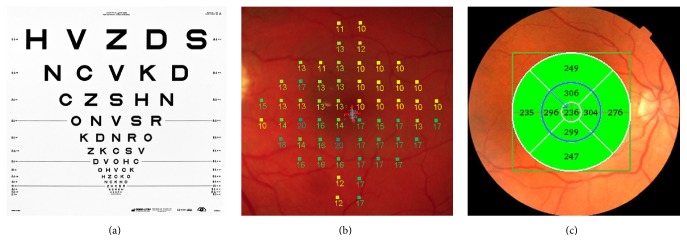
(a) ETDRS chart for best corrected visual acuity test. (b) Macular photography with the microperimetry test area. (c) Optical coherence tomography of macula and ETDRS rings thickness measurements, blue arrow to the central subfield, encircled blue central 5-field area.

Preoperative visit evaluation in addition to routine comprehensive ophthalmological exam included best corrected visual acuity using the ETDRS charts (4-meter 2000 series revised ETDRS chart (Precision Vision®, La Salle, USA)), measurement of retina sensitivity by microperimetry (MP-1 Micro Perimeter, Nidek), and measurement of macular thickness, using optical coherence tomography (Topcon 3D OCT-1000, Tokyo, Japan) (Figure 1).

At all follow-up visits, the complete ophthalmological exam with best corrected visual acuity using the ETDRS charts and the optical coherence tomography of the macula were repeated. Additionally, microperimetry was repeated at 1, 3, and 6 months after surgery.

The scanning protocol for optical coherence tomography used in this study was the Fast Macular Thickness program (Topcon 3D OCT-1000, Tokyo, Japan), which creates a retinal map algorithm consisting of six radiating cross-sectional scans, each of 6 mm length, that produces a circular plot in which the fovea is a central circular zone of 1 mm diameter. Superior, nasal, inferior, and temporal parafoveal zones represent annular bands in these respective sectors. There are other two concentric zones, the first having a diameter of 3 mm and the second one of 6 mm. The nine zones (the central zone is named field 1, the first annular ring fields 2–5, and the second annular ring fields 6–9) have been called ETDRS-type regions because of their similarity to zones of analysis of photographs by ETDRS graders (Figure 1(c)) [20, 21]. To correlate retinal thickness data accurately with retinal sensitivity data, we compared the central fields 1 to 5, and we excluded fields 6 to 9, as the microperimetry MP-1 grid covered only a limited area of these latter fields (Figures 1(b) and 1(c)).

Microperimetry was performed after pupil dilatation with automatic fundus-related perimeter (MP-1 Micro Perimeter; Nidek Technologies, Padova, Italy) (Figure 1(b)) [22]. The fundus is imaged in real time; the fixation target and stimuli are projected onto the retina. The central 10° visual field was tested with the Humphrey 10–2°16 dB56s program, fast strategy, and background illumination: 1.27 cd/m^2^, stimulation time 200 ms, stimulation spot size: Goldmann III. In all, 0 dB (equivalent to 1.27 cd/m^2^) represented the brightest luminance, and the stimulus intensity varied from 0 to 20 dB. The subsequent exams of the same eye were performed using the follow-up option of the software, which enables projection of the testing spots at the exact same area of the retina.

### 2.4. Statistical Analysis

The statistical analysis of the data was performed using SPSS (SPSS, Inc., Chicago, IL) for Windows 11.5 package program. Power analysis was performed to detect the sample size. Mean standard deviation (SD) was used to describe quantitative data. Student's *t*-test was used to analyze the difference between two samples and ANOVA was used for analysis of more samples together in macular sensitivity and thickness in the two groups. Threshold of statistical significance was 0.05.

## 3. Results

Twenty-eight eyes of twenty-eight patients, with a mean age of 71.1 ± 6.9 (SD) years, underwent cataract surgery. Participants had a clinical diagnosis of cataract and diabetes, diabetes mellitus type 2 without diabetic retinopathy (*n* = 18), mean age 73.5 ± 7.01 (SD) or cataract and no diabetes (*n* = 10), mean age 68.8 ± 4.70 (SD); the age difference was not statistically significant, *p* = 0.167. The accompanying systemic diseases are presented in Table 1.

The cataract surgery was uneventful in all eyes. The mean phacoemulsification time was 2,73 seconds, without statistically significant difference between the groups.

### 3.1. Visual Acuity

**Figure 2:**
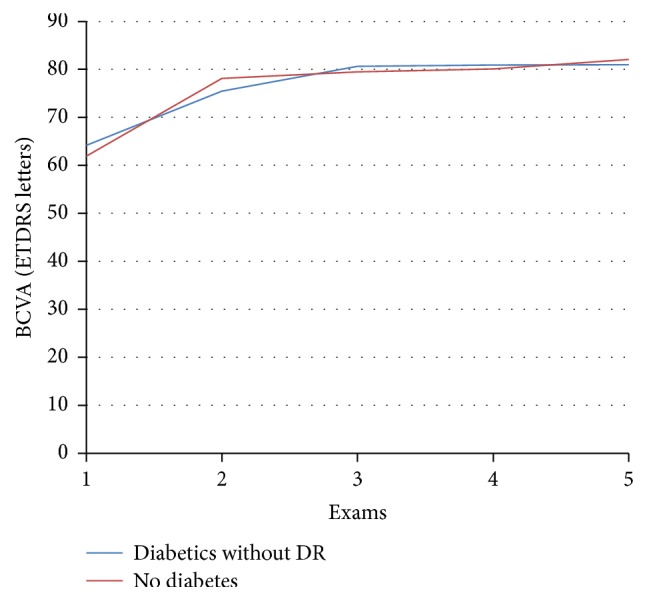
Best corrected visual acuity (BCVA) in ETDRS letters before (exam 1) and after cataract surgery: 1st day (exam 2), 1 month (exam 3), 3 months (exam 4), and 6 months (exam 5).

Table 2 and Figure 2 show the mean best corrected visual acuity (BCVA) in diabetics without DR and the no-diabetes group eyes at baseline, day 1, 1 month, 3 months, and 6 months after cataract surgery. BCVA improved in both groups.

The mean best corrected visual acuity ETDRS in the diabetics without diabetic retinopathy group eyes improved for 16.8 ETDRS letters or 26.2%, *p* < 0.001. Equivalently, in the no-diabetes group eyes, it improved for 20.2 ETDRS letters or 32.6%, *p* < 0.001, without significant difference between the groups (*p* = 0.183). All eyes achieved a BCVA of 76 or more and 75 or more ETDRS letters in the diabetic and control group, respectively.

### 3.2. Optical Coherence Tomography Thickness Measurements

**Figure 3:**
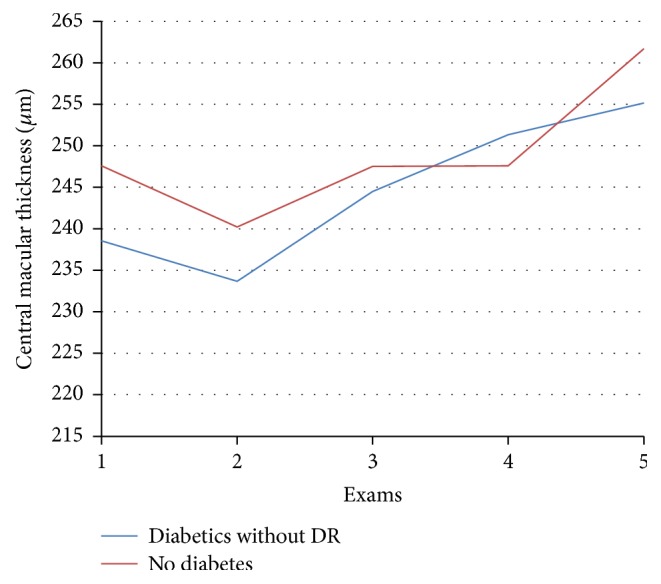
Central macular thickness on optical coherence tomography in *μ*m before (exam 1) and after cataract surgery: 1st day (exam 2), 1 month (exam 3), 3 months (exam 4), and 6 months (exam 5).

At OCT examination, mean retinal thickness in the central field (field 1) in the diabetic group changed from 238.6 *μ*m preoperatively to 255.2 (*p* = 0.02) 6 months after cataract surgery. In the control group, the thickness in the central field changed from 247.6 *μ*m preoperatively to 261.7 (*p* = 0.03) 6 months after cataract operation (Table 3, Figure 3).

**Figure 4:**
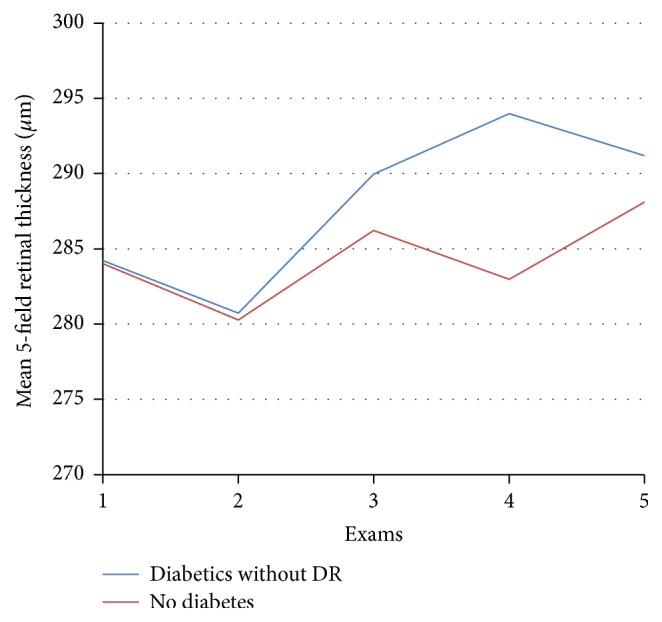
Mean retinal thickness of the macular 5 fields on optical coherence tomography in *μ*m before (exam 1) and after cataract surgery: 1st day (exam 2), 1 month (exam 3), 3 months (exam 4), and 6 months (exam 5).

**Figure 5:**
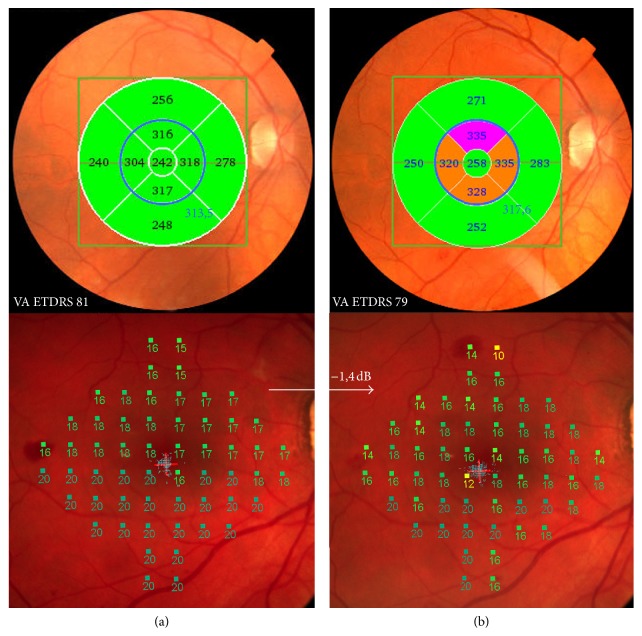
Investigations of a 58-year-old diabetic patient. (a) One month postoperatively: above mean thickness in the central 5-field macular area at OCT (blue circle), BCVA, and below microperimetry sensitivity. (b) Six months postoperatively: above increased mean thickness in the central 5-field macular area at OCT (blue circle), decreased number of ETDRS letters of BCVA, and below microperimetry mean sensitivity decrease (arrow).

Observing a wider macular area, namely, the central five fields (Figure 5), the mean retinal thickness in diabetics without DR showed increased thickness from the preoperative value of 284.2 to 291.2 *μ*m 6 months after surgery (*p* = 0.03) (Table 4). In the control group, however, the values did not show significant changes (Table 4, Figure 4).

### 3.3. Microperimetry

Mean sensitivity on microperimetry showed relatively stable improvement in the diabetic group when comparing preoperative exam to 1-, 3-, and 6-month postoperative follow-up exams; the average difference was 3.62 dB (SD 2.31), 3.69 (SD 2.39), and 3.45 (SD 2.34). Direct comparison of the mean sensitivity improvement after cataract surgery between the DM2 without DR and the control eyes groups was not done, because the absolute improvement depends mainly on the severity of the cataract, and the groups were not balanced on the basis of cataract severity. However, when the comparison was made among the postoperative follow-up exams between the groups, a decrease in the mean sensitivity was found in the DM2 without DR group eyes (Figure 5) but not in the control group eyes. In the DM2 without DR group eyes, a mean average difference of −0.75 dB (SD 2.38) from 1 to 6 months of follow-up was observed. The control group eyes, on the other hand, showed increased mean sensitivity of 1.3 dB (SD 2.13) when compared to the postoperative follow-up exams from 1 to 6 months after surgery.

The microperimetry testing area corresponds to the five-field retina on the OCT, so the above results were paralleled and correspondent.

## 4. Discussion

In our study, we demonstrated that cataract surgery in diabetic patients without diabetic retinopathy did not influence the good visual acuity outcome after cataract surgery. However, slight increase in paracentral macular thickness and a corresponding decrease in mean retinal sensitivity after 6 months were present in diabetics without diabetic retinopathy group eyes and not in the control group.

Our study included both functional and morphological examination methods of the macula, in order to try to find clinical and subclinical difference after cataract surgery between nondiabetic and diabetic patients without diabetic retinopathy. A wider macular area was examined additional to the central macular thickness, and macular sensitivity test was added to the visual acuity testing, which, according to our knowledge, was not analyzed in previous published studies.

In our study, both groups, diabetics and nondiabetic patients, showed equal statistically significant thickening of the central macular subfield 6 months after cataract operation, diabetics for 16.6 *μ*m and controls for 14.1 *μ*m after 6 months. Cataract surgery is a source of oxidative stress, which can result in macular edema, as it produces free radicals, causes medium termed lowering of antioxidative substances in the anterior chamber, and also influences normal oxygen levels in the eye on the long term by removing lens epithelial cells [7, 23]. The production of free radicals can be influenced by the cataract grade, as more phaco energy is usually used, any possible complications during the surgery, and longer operation time for any other reasons. All of these factors were controlled in the study to prove if diabetes is a factor of difference.

Additional analysis of the ETDRS subfields at OCT of the macula showed slightly more pronounced thickening in the cumulative central 5 fields in the diabetic group 6 months after surgery in comparison to the control group. The area covered by central 5 fields corresponds to the testing area of the automated microperimetry retinal sensitivity testing [24]. A matching decrease in mean retinal sensitivity at 6 months was found in the diabetic group. Microperimetry added to visual acuity and thickness measurements shows a supplementary macular function, especially of value in patients at risk for macular changes. Cataract surgery itself plays an important risk factor for macular thickening and also for occurrence of macular edema with vision deterioration by releasing prostaglandins and increasing oxidative stress [23]. Because of this additional oxidative stress caused by cataract surgery in diabetic patients, who are already exposed to higher oxidative stress due to the underlying disease, macular thickening could be expected to occur more often and to be more exaggerated.

Several authors reported increased central macular thickness in diabetic patients with diabetic retinopathy after cataract surgery. Kim et al. studied changes in central point thickness on optical coherence tomography (OCT) after uncomplicated cataract operation in diabetic patients with different status of the retina and reported thickening for more than 30% in 22% of the participants [16]. Kwon et al. reported that after cataract surgery 18% of diabetic patients with diabetic retinopathy developed thickening of more than 30% of the central subfield of the macula, which correlated to the severity of retinopathy [17]. The risk for macular thickening after cataract surgery depends on the severity of retinopathy and/or preexisting diabetic macular edema (DME) [13, 16]. However, a meta-analysis report claimed no significant difference in central macular thickness 1 and 3 months after cataract surgeries in diabetic patients without diabetic retinopathy [12]. In terms of prevention of postoperative worsening of DME and improvement of the final visual outcome, it is advisable to inject an intravitreal anti-VEGF drug during cataract operation in patients with preexistent diabetic macular edema [13, 25, 26], and there are clinical studies done proving that even patients with stable diabetic retinopathy without significant macular edema do benefit from this procedure in terms of less thickening of the macula and better visual outcome [13, 27].

Our results show an increase in the central 5-field area of macular thickness and a corresponding decrease in retinal sensitivity in diabetic patients without diabetic retinopathy 6 months after cataract surgery not observed in the control group and not reported before. Both OCT measurements and microperimetry are known to show diurnal and long term variability; thus, the results need to be interpreted carefully [28–31]. In our study, the differences between diabetic patients without retinopathy and nondiabetic patients were observable, but they were small and subclinical and could only represent a greater magnitude of variability connected to pathologic conditions [32]. On the other hand, several new and precise measures available today do offer new data, which might be proved useful and important in the future. And the changes observed in diabetic patients in the study might be important in predicting possible changes over a longer time period.

There are certain limitations of this study. There were a relatively small number of cases included, and the optical coherence tomography measurements were not repeated.

In conclusion, the combination of increased macular thickness in the central 5-field macular area and decreased retinal sensitivity in diabetics after cataract surgery in the era of premium intraocular lenses and refractive lens procedures, where a perfect status of the macula is desired, might be of interest. On the other hand, the improvement of the best corrected visual acuity and only minimal functional macular changes ease the decision to perform cataract surgery in diabetic as in healthy patients when appropriate. However, diabetics should be treated with additional caution, the use of topical NSAID after cataract surgery should be considered, and follow-up visits could be more accurate with additional measurements of macular morphology and function.

Whether changes in macular thickness and retinal sensitivity observed in diabetic patients without diabetic retinopathy in our study could progress to clinical important consequence over a longer period of time remains to be elucidated in studies with longer follow-up and higher number of subjects enrolled.

## Figures and Tables

**Table 1:** Demographics of all patients included in the study with type of diabetes treatment and percentage of accompanying systemic diseases.

	Number of eyes	Age (years)	Duration of diabetes (years)	Therapy for diabetes (% of patients)	Systemic diseases (% of patients)
Diabetics without DR	18	57–83 mean 73.5 SD 7.01	1–30 mean 11.8 SD 8.6	Insulin (17%) Per os (75%) Diet (8%)	Arterial hypertension (83%) Hyperlipidemia (8%)

No diabetes	10	60–73 mean 68.8 SD 4.70	NA	NA	Arterial hypertension (40%) Hyperlipidemia (20%)

		*p* = 0.167^*∗*^			

NA: not applicable.

^*∗*^Significance level *p* < 0.05.

**Table 2:** Mean best corrected visual acuity (BCVA) in ETDRS letters preoperatively and at all postoperative follow-ups.

	BCVA					The association between preoperative and postoperative (6 months) BCVA
	Preoperative	Postoperative				
		Day 1	1 month	3 months	6 months	
Diabetics without DR	64.2 ± 5.6 (SD)	75.5 ± 5.9 (SD)	80.7 ± 3.6 (SD)	80.9 ± 3.9 (SD)	81.0 ± 2.9 (SD)	*p* < 0.001
No diabetes	61.9 ± 8.9 (SD)	78.1 ± 3.9 (SD)	79.5 ± 3.5 (SD)	80.1 ± 2.1 (SD)	82.1 ± 3.7 (SD)	*p* < 0.001

Significance level if *p* < 0.05.

**Table 3:** Central macular thickness (CMT) in *µ*m at optical coherence tomography preoperatively and at all follow-ups.

	CMT (*µ*m)					The association between preoperative and postoperative (at 6 months) BCVA
	Preoperative	Postoperative				
		Day 1	1 month	3 months	6 months	
Diabetics without DR	238.6 ± 29.0 (SD)	233.7 ± 30.2 (SD)	244.5 ± 24.0 (SD)	251.3 ± 27.8 (SD)	255.2 ± 31.5 (SD)	*p* = 0.02
No diabetes	247.6 ± 25.0 (SD)	240.2 ± 23.0 (SD)	247.5 ± 21.0 (SD)	247.6 ± 20.0 (SD)	261.7 ± 29.0 (SD)	*p* = 0.03

Significance level if *p* < 0.05.

**Table 4:** Mean retinal thickness of the macular 5 fields (RT 5 fields) at optical coherence tomography in *µ*m preoperatively and at all follow-ups.

	RT 5 fields (*µ*m) preoperative	RT 5 fields Day 1	RT 5 fields 1 month	RT 5 fields 3 months	RT 5 fields 6 months	The association between preoperative and postoperative (6 months) OCT RT 5 fields
Diabetics without DR	284.2 ± 19.1 (SD)	280.7 ± 19.1 (SD)	290 ± 19.4 (SD)	294 ± 16.7 (SD)	291.2 ± 16.8 (SD)	*p* = 0.03
No diabetes	284.0 ± 18.0 (SD)	280.3 ± 18.0 (SD)	286.2 ± 15.0 (SD)	283 ± 13.0 (SD)	288.1 ± 20.0 (SD)	*p* = 0.32

Significance level if *p* < 0.05.
